# Supplementary material for: Rescued from extinction in two buckets: genomic consequences of an extreme bottleneck in the endangered Owens pupfish
Source: bioRxiv. 2026 Oct 28:2026.10.26.761965. Preprint. [Version 1] doi: 10.64898/2026.10.26.761965 (PMC13733973; doi:10.64898/2026.10.26.761965)
Supplement: Supplementary file 1 [file media-1.pdf]

Supporting information for

Rescued from extinction in two buckets: genomic consequences of an extreme bottleneck in the endangered Owens pupfish

David Tian, Bruce J. Turner, Christopher H. Martin

Corresponding author: David Tian

Email: dtian@ucla.edu

This file includes:

Supplementary Figures S1 - S2

Supplementary Tables S1 - S2

Supplemental Figures

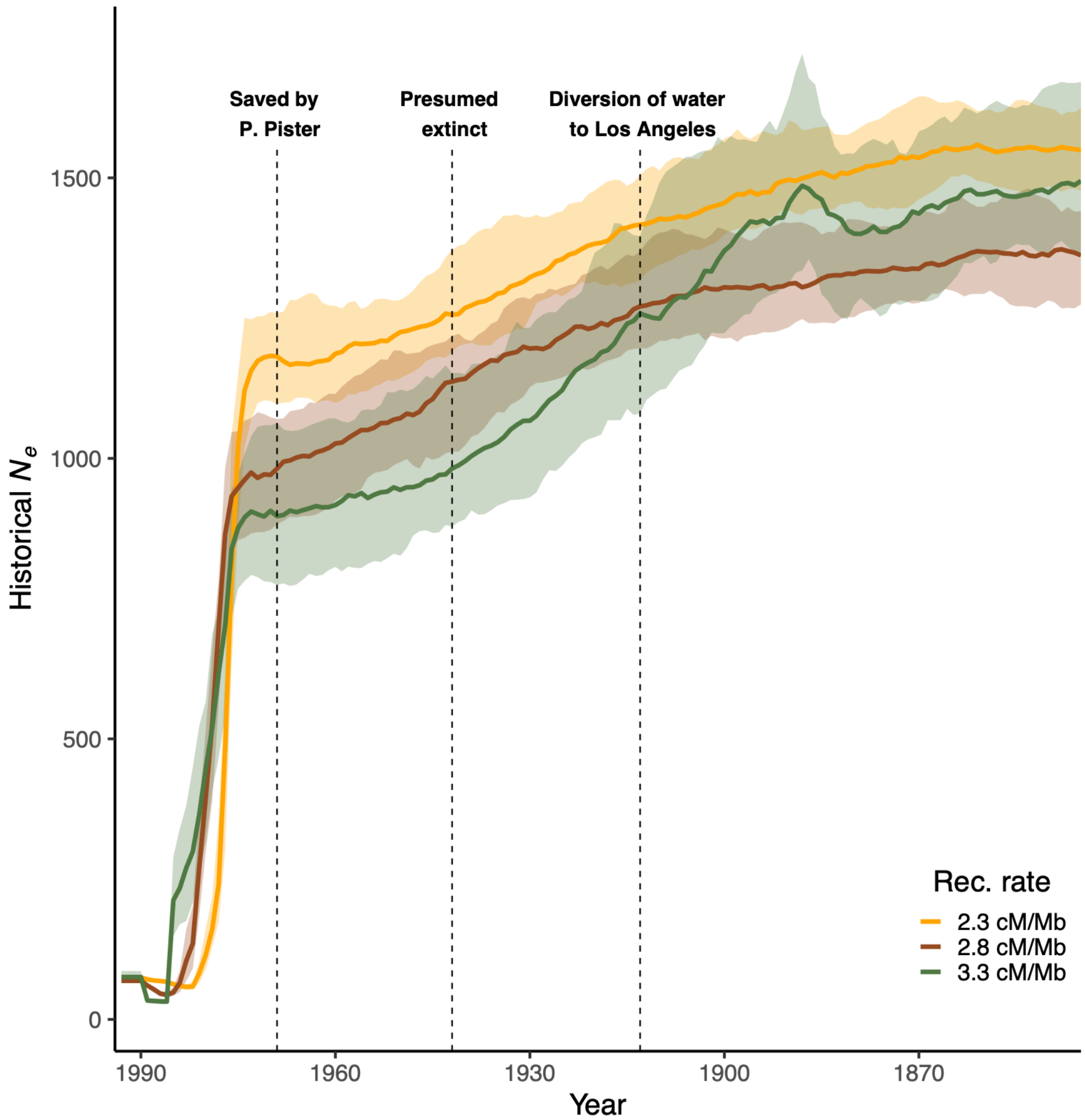

Supp. Fig. 1. Recent effective population size of Owens pupfish over the past 150 generations estimated with GONE2 (Santiago et al. 2025) based on 10 individuals from the White Mountain Research Station population. Here, we assume that generation time is one year. Colors represent varying recombination rates used to parameterize GONE2 and shaded areas represent the 95% confidence interval across 50 bootstraps. Dashed vertical lines mark when water was diverted from the Owens River to Los Angeles (1913), when Owens pupfish were presumed extinct (1948), and when P. Pister saved the species in two buckets (1969).

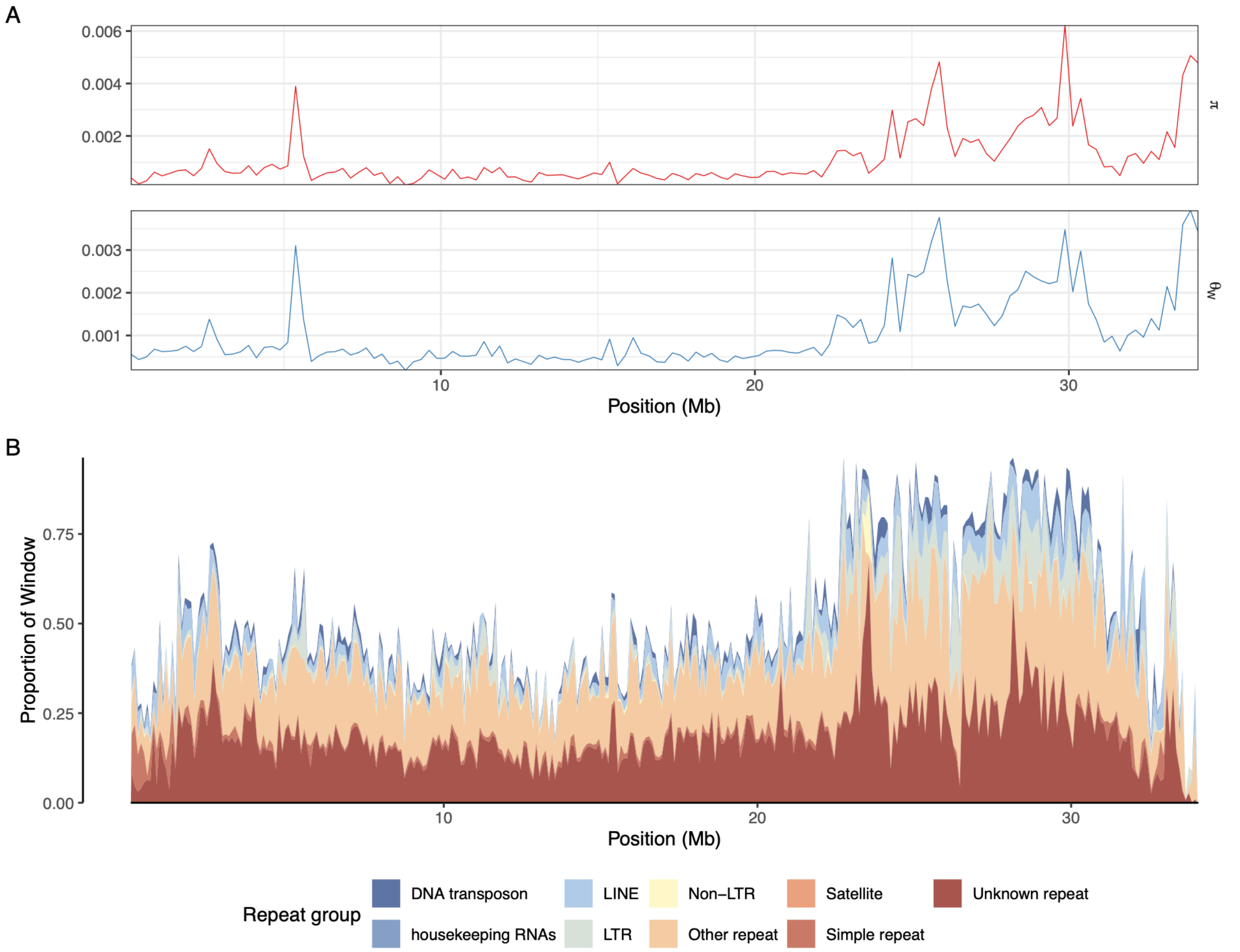

Supp. Fig. 2. Genetic diversity along chromosome 24. A) Nucleotide diversity and Watterson's theta in non-overlapping sliding windows of 250 kb calculated with pixy (Korunes and Samuk 2021). B) Distribution of repeat content based on RepeatMasker annotations of the *Cyprinodon nevadensis mionectes* reference genome (GCA_030533455.1).

Supplemental Tables

Supp. Table 1. Known genes found in the upper 99th percentile of nucleotide diversity outlier windows. Gene functions sourced from GeneCards.

Gene ID	Gene name / function
ALOX12B	arachidonate 12-lipoxygenase, 12R type
AMICA1	enables integrin binding activity and protein homodimerization activity
CD84	SLAM family self-ligand receptor and membrane glycoprotein
DOCK3	dedicator of cytokinesis family guanine nucleotide exchange factor
Dref	DNA replication-related element factor
FCRLA	Fc receptor-like A, an immunoglobulin-like protein related to receptors for the Fc fragment of gamma immunoglobulin G.
HAUS7	subunit of the augmin complex that regulates centrosome and mitotic spindle integrity
L1TD1	LINE-1 Type Transposase Domain-Containing Protein 1
MAP6	microtubule-associated protein 6
MOG	myelin oligodendrocyte glycoprotein
NLRP12	cytoplasmic NOD-like receptor that attenuates inflammation in activated monocytes
NOL9	nucleolar protein with ATP-dependent polydeoxyribonucleotide 5'-hydroxyl-kinase activity
PRKRIR	Protein-Kinase, Interferon-Inducible Double Stranded RNA Dependent Inhibitor
SLC25A11	mitochondrial dicarboxylate carrier that mediates electroneutral exchange of 2-oxoglutarate for malate and other dicarboxylic acids across the inner mitochondrial membrane
SPTLC2	catalytic subunit of serine palmitoyltransferase that initiates sphingolipid biosynthesis
TARP	TCR Gamma Alternate Reading Frame Protein
TCP11L1	T-Complex 11 Like 1
TMEM185A	transmembrane protein 185A, a predicted membrane protein that localizes to the CpG island of the fragile site FRAXF
TRAV18	variable region of the T cell receptor alpha chain that contributes to antigen recognition in alpha-beta T cell receptors and supports adaptive immunity
TRAV21	ariable region of the T cell receptor alpha chain that contributes to antigen recognition within peptide–major histocompatibility complexes and helps initiate T cell activation
TRIM7	E3 ubiquitin-protein ligase and member of the tripartite motif (TRIM) family that links selective substrate recognition to ubiquitin-mediated protein degradation
ZDHHC24	ZDHHC palmitoyltransferase 24, a DHHC-type enzyme predicted to catalyze protein-cysteine S-palmitoyltransferase activity
ZMYM6	zinc finger MYM-type protein associated with DNA binding and roles in cytoskeleton organization and regulation of cell morphogenesis
ZNF251	KRAB-domain zinc finger transcription factor that binds DNA and regulates RNA polymerase II-dependent transcription
ZNF846	zinc finger protein 846, a KRAB domain-containing C2H2 zinc-finger transcription factor predicted to bind DNA and regulate RNA polymerase II-dependent transcription
wrn-1	multifunctional RecQ helicase-exonuclease that preserves genomic integrity through ATP-dependent 3'→5' helicase and 3'→5' exonuclease activities

Supp. Table 2. Individual sample metadata. Location abbreviation of WMRS represents White Mountain Research Station. Genome-wide mean coverage and mapping quality were calculated using qualimap (v2.2.2) and individual missingness was calculated with vcftools (v0.1.16).

Sample ID	Population	Sample Year	Mean coverage	Mapping quality	Ind. missingness
CRAD_18-10	WMRS	1994	22.27X	21.96	0.062
CRAD_18-11	WMRS	1994	24.61X	22.22	0.056
CRAD_18-2	WMRS	1994	16.96X	21.73	0.083
CRAD_18-3	WMRS	1994	14.47X	21.38	0.124
CRAD_18-4	WMRS	1994	18.98X	21.68	0.077
CRAD_18-5	WMRS	1994	18.90X	22.19	0.078
CRAD_18-6	WMRS	1994	16.93X	22.13	0.096
CRAD_18-7	WMRS	1994	13.74X	21.94	0.129
CRAD_18-8	WMRS	1994	17.27X	21.90	0.082
CRAD_18-9	WMRS	1994	21.20X	21.90	0.064
CYPRAD18_1	WMRS	1994	7.18X	21.59	0.458
C_radiosus_W368-2	Well 368	2010	12.86X	21.15	0.159
C_radiosus_W368-8	Well 368	2010	12.45X	20.96	0.178
